# Supplementary material for: Effects of histamine on human periodontal ligament fibroblasts under simulated orthodontic pressure
Source: PLoS One. 2020 Aug 7;15(8):e0237040. doi: 10.1371/journal.pone.0237040 (PMC7413485; doi:10.1371/journal.pone.0237040)
Supplement: S1 Fig — (DOCX) [file pone.0237040.s001.docx]

**S1 Fig. Fold changes in COX-2 and RANKL gene expression** **by PDLF** due to pressure application for 48 h for each individual subject included into the used pool of PDLF.
