# Supplementary material for: Effects of histamine on human periodontal ligament fibroblasts under simulated orthodontic pressure
Source: PLoS One. 2020 Aug 7;15(8):e0237040. doi: 10.1371/journal.pone.0237040 (PMC7413485; doi:10.1371/journal.pone.0237040)
Supplement: S2 Fig — Effects of histamine and 50 μM H1R antagonist fexofenadine (F9427, Sigma-Aldrich) on COX-2 (a) and IL-6 (b) gene expression. AU: arbitrary units; *p ≤ 0.05; ** p ≤ 0.01. Statistics: Welch-corrected ANOVA with Games-Howell posthoc tests. Each symbol in figures represents a data point. Horizontal lines represent the mean ± standard error of mean. (DOCX) [file pone.0237040.s002.docx]

**
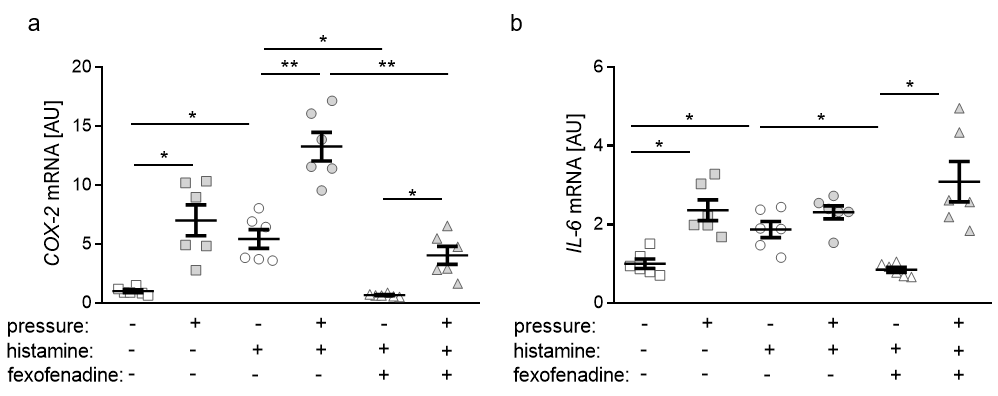
 S2 Fig. Effects of histamine and 50 µM H1R antagonist fexofenadine (F9427, Sigma-Aldrich) on *COX-2* (a) and *IL-6* (b) gene expression.** AU: arbitrary units; *p ≤ 0.05; ** p ≤ 0.01. Statistics: Welch-corrected ANOVA with Games-Howell posthoc tests. Each symbol in figures represents a data point. Horizontal lines represent the mean ± standard error of mean.
