# Supplementary material for: Effects of histamine on human periodontal ligament fibroblasts under simulated orthodontic pressure
Source: PLoS One. 2020 Aug 7;15(8):e0237040. doi: 10.1371/journal.pone.0237040 (PMC7413485; doi:10.1371/journal.pone.0237040)

**Uncropped agarose gel image presented in Fig. 2**

H1R


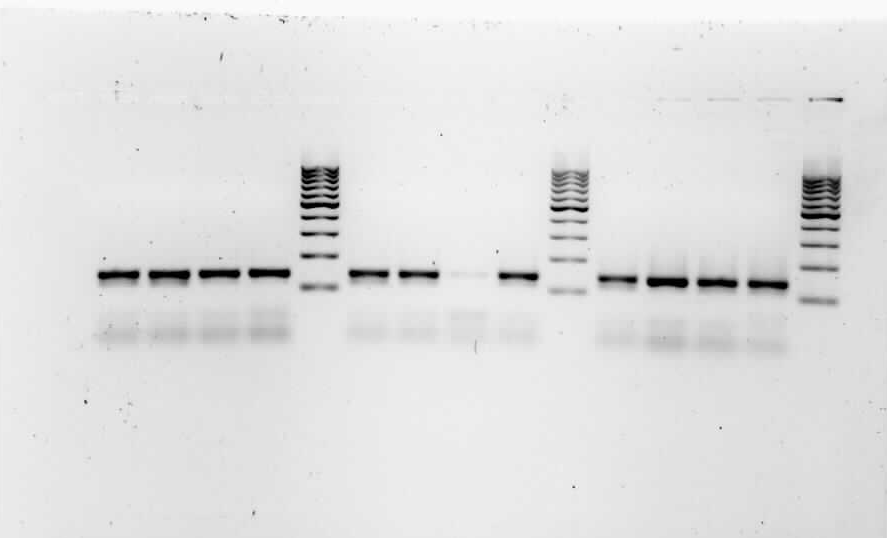


H2R

**
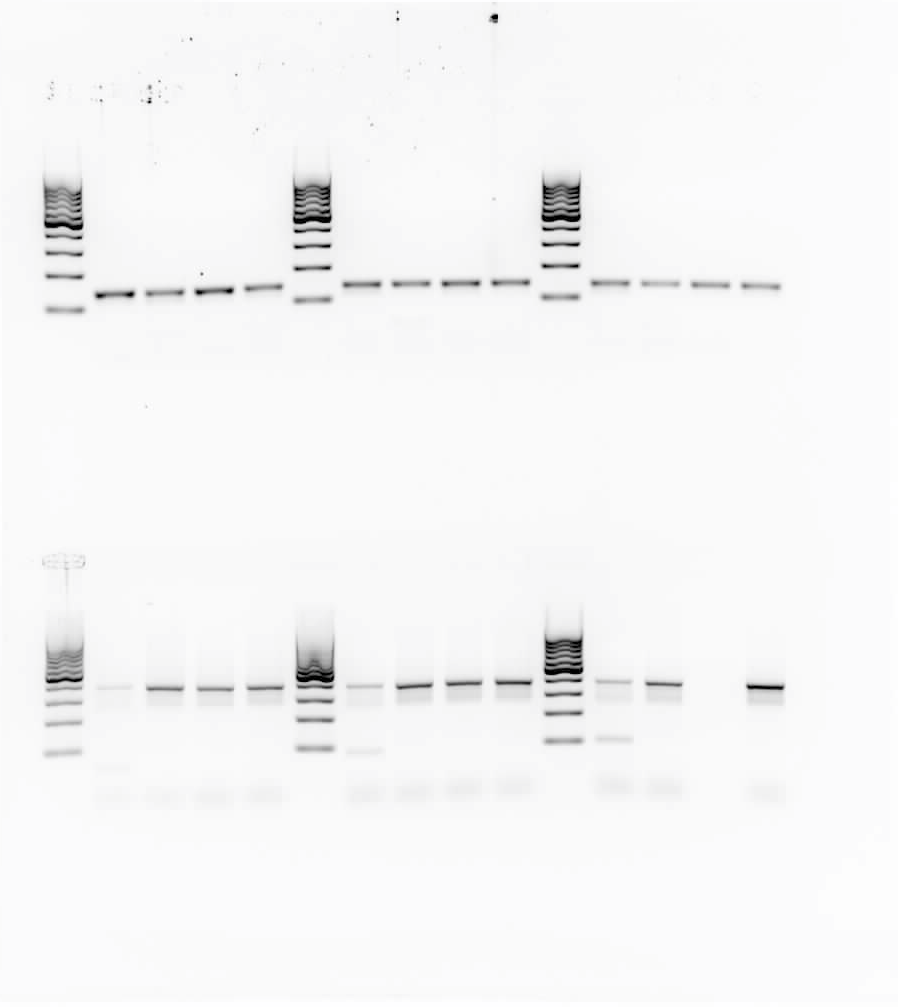
**

H3R (left) and H4R (right)

**
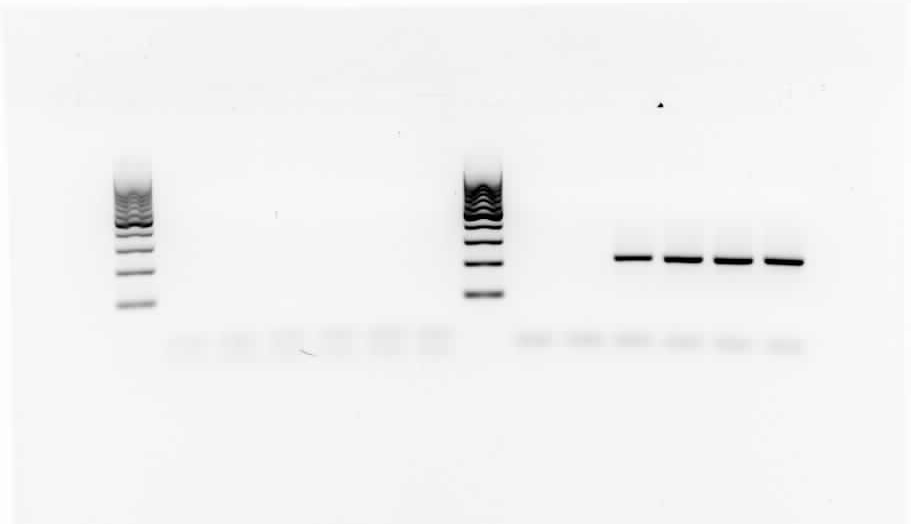
**

RPL22

**
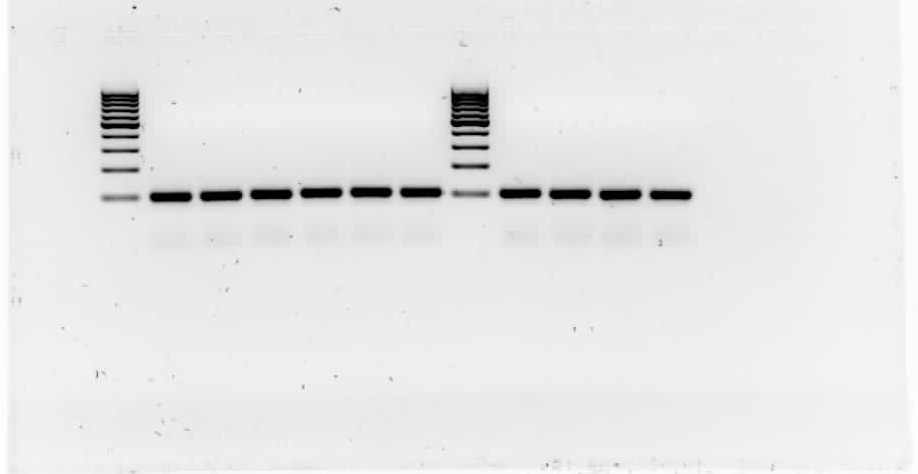
**

**Uncropped Western Blot image presented in Fig. 5**


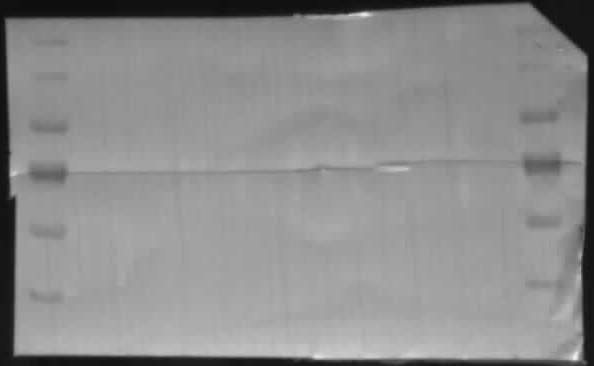

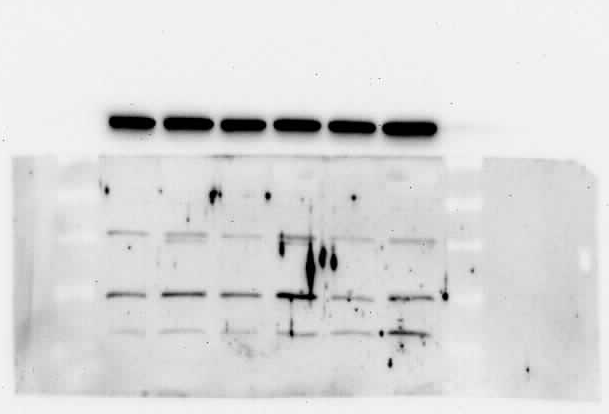


**Original data information**

**Fig. 2**


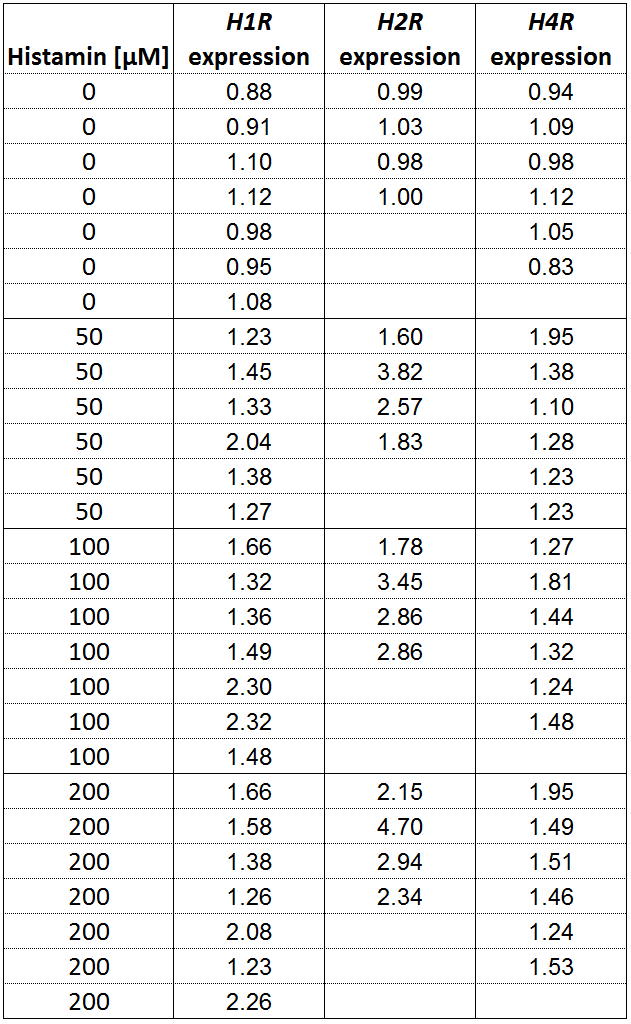


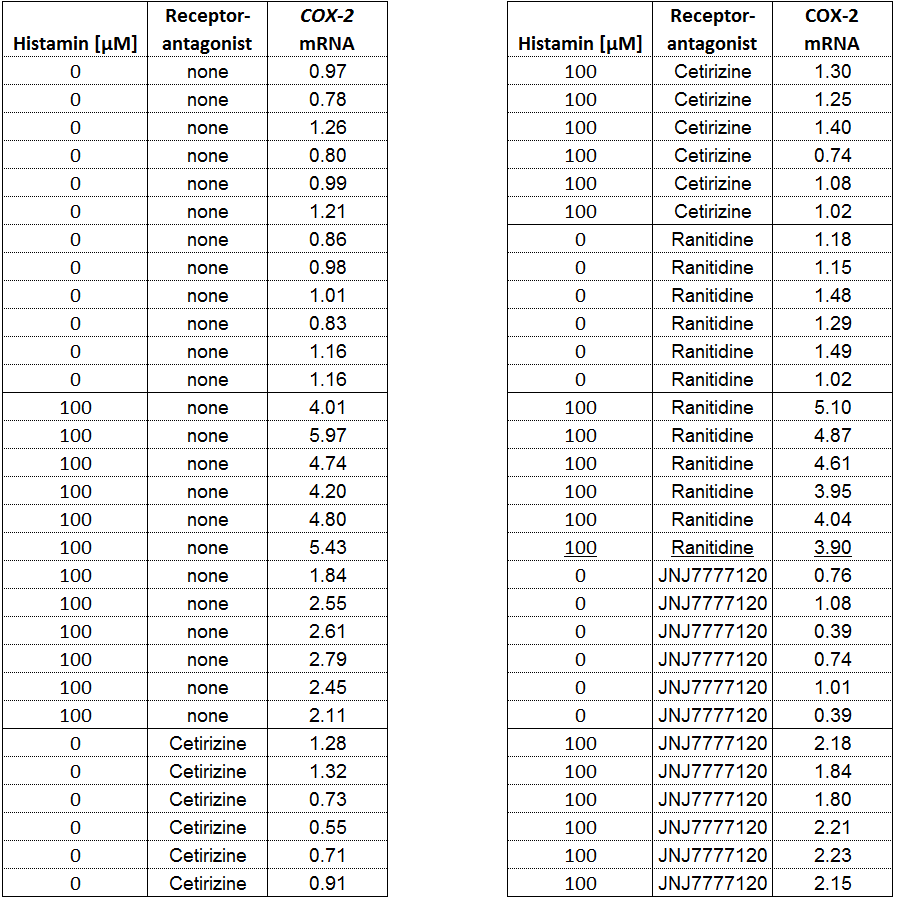


**Fig. 3**


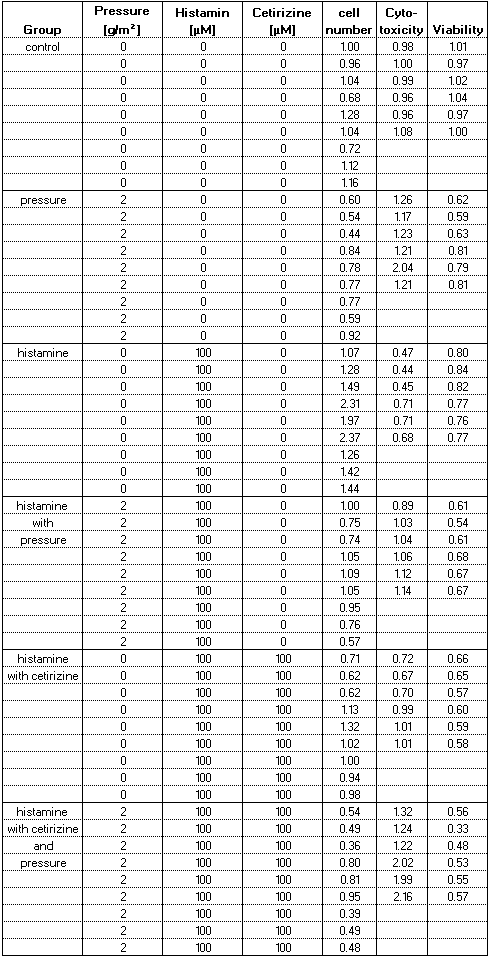


**Fig. 4**


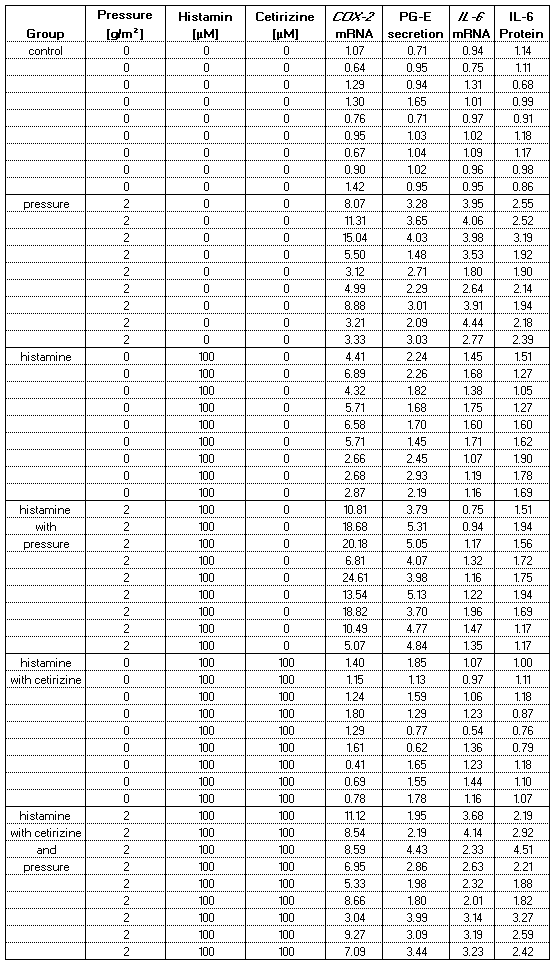


**Fig. 5**


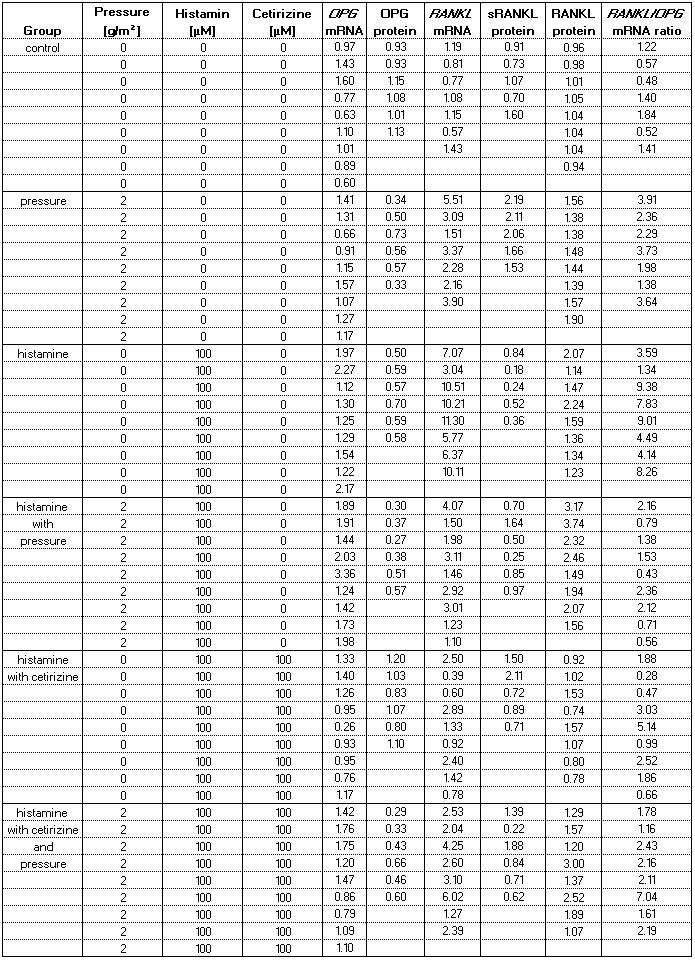

Supplement: S1 Raw Images — (DOCX) [file pone.0237040.s004.docx]
